# Increase in Bone Mineral Density of the Amputated-Side Femur Following High-Impact Training in a Young Transtibial Amputee: A Case Report

**DOI:** 10.7759/cureus.96483

**Published:** 2025-11-10

**Authors:** Mitsunori Toda, Takaaki Chin, Takashi Oshima

**Affiliations:** 1 Rehabilitation Medicine, Hyogo Rehabilitation Center Hospital, Kobe, JPN; 2 Robot Rehabilitation Center, The Hyogo Institute of Assistive Technology, Kobe, JPN; 3 Orthopaedic Surgery, Hyogo Rehabilitation Center Hospital, Kobe, JPN

**Keywords:** bone mineral density, case report, dual-energy x-ray absorptiometry (dxa), high impact exercise, lower limb amputation, lower-limb prosthesis, physical medicine and rehabilitation, transtibial amputation

## Abstract

Loss of bone mineral density (BMD) in the amputated limb is a well-documented complication after lower limb amputation, predisposing patients to osteoporosis and fragility fractures. Exercise interventions have been recommended to mitigate this decline, but to date no clinical reports have demonstrated an actual increase in hip BMD following amputation.

We describe a young male dockworker who underwent transtibial amputation after severe occupational trauma. A structured rehabilitation program incorporating high-impact and resistance training was delivered for four months during hospitalization, followed by continued self-directed exercise after discharge. Serial dual-energy X-ray absorptiometry (DXA) scans over three years revealed progressive gains in amputated-side hip BMD, with final measurements showing a 17.4-24.3% increase from baseline.

This appears to be the first longitudinally documented case of improved hip BMD in an amputated limb following structured rehabilitation with high-impact exercise. The program closely paralleled recent expert recommendations on exercise prescription for amputees, underscoring the potential of targeted progressive loading to preserve skeletal health. However, the relative contribution of exercise intensity versus other factors remains uncertain, and further studies are needed to establish dose-response relationships and generalizability.

## Introduction

Lower limb amputation is associated with substantial bone mineral density (BMD) loss in the amputated limb, particularly at the proximal femur [1-3]. Factors contributing to this loss include reduced mechanical loading, altered gait patterns, and disuse atrophy [3-5]. BMD reduction is more pronounced with proximal amputation levels but is also observed after transtibial amputation [4].

Such loss occurs early after amputation and often persists even after prosthetic gait training is achieved [6]. This reduction in hip BMD is clinically significant, as it increases the risk of osteoporosis and hip fractures, which have an estimated incidence of 2.35-3.8% in amputees [7,8]. Hip fractures in this population not only impair mobility but also impose considerable socioeconomic burdens [9].

In other populations, such as postmenopausal women [10], older men [11,12], patients with anorexia nervosa [13], and astronauts in microgravity [14], targeted exercise interventions have been shown to increase or preserve hip BMD. Recently, a Delphi consensus study proposed specific exercise recommendations to minimize hip BMD loss after traumatic lower limb amputation [15], but their efficacy has not been validated in clinical cases.

To our knowledge, no previous reports have demonstrated an actual increase in hip BMD after lower limb amputation. We report a case of a young transtibial amputee who achieved a substantial increase in amputated-side proximal femoral BMD following a rehabilitation program incorporating high-impact exercise.

## Case presentation

A male dockworker in his early twenties sustained a severe left foot injury after his foot was caught in heavy machinery. Initial management at a tertiary trauma center included partial foot amputation, surgical debridement, pin fixation, and subsequent skin grafting. Despite multiple surgical interventions, persistent infection was observed, accompanied by progressive plantarflexion contracture of the ankle.

**Figure 1:**
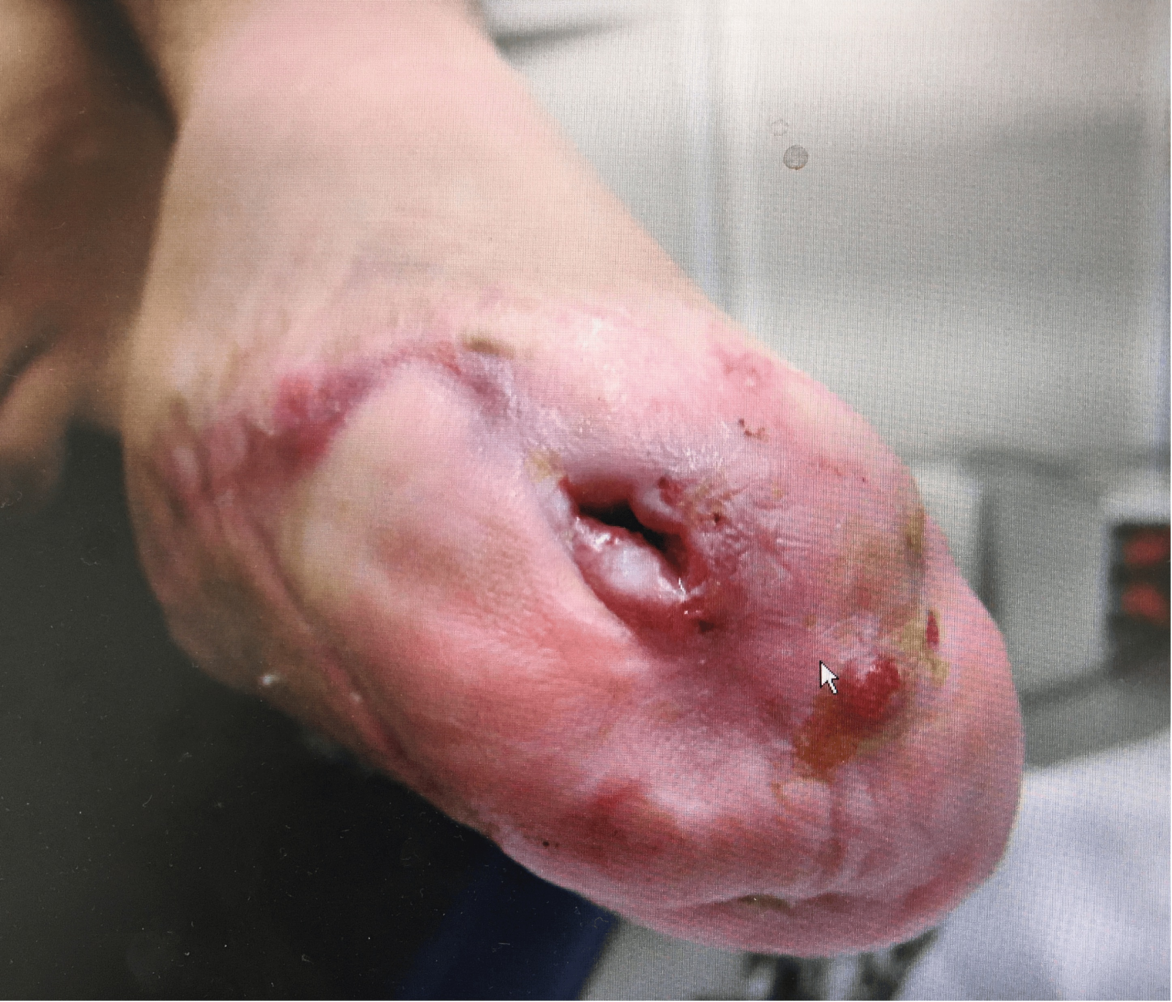
Clinical photograph of the residual foot at the time of initial presentation Clinical image illustrating a sinus tract with persistent drainage and fixed ankle deformity.

**Figure 2:**
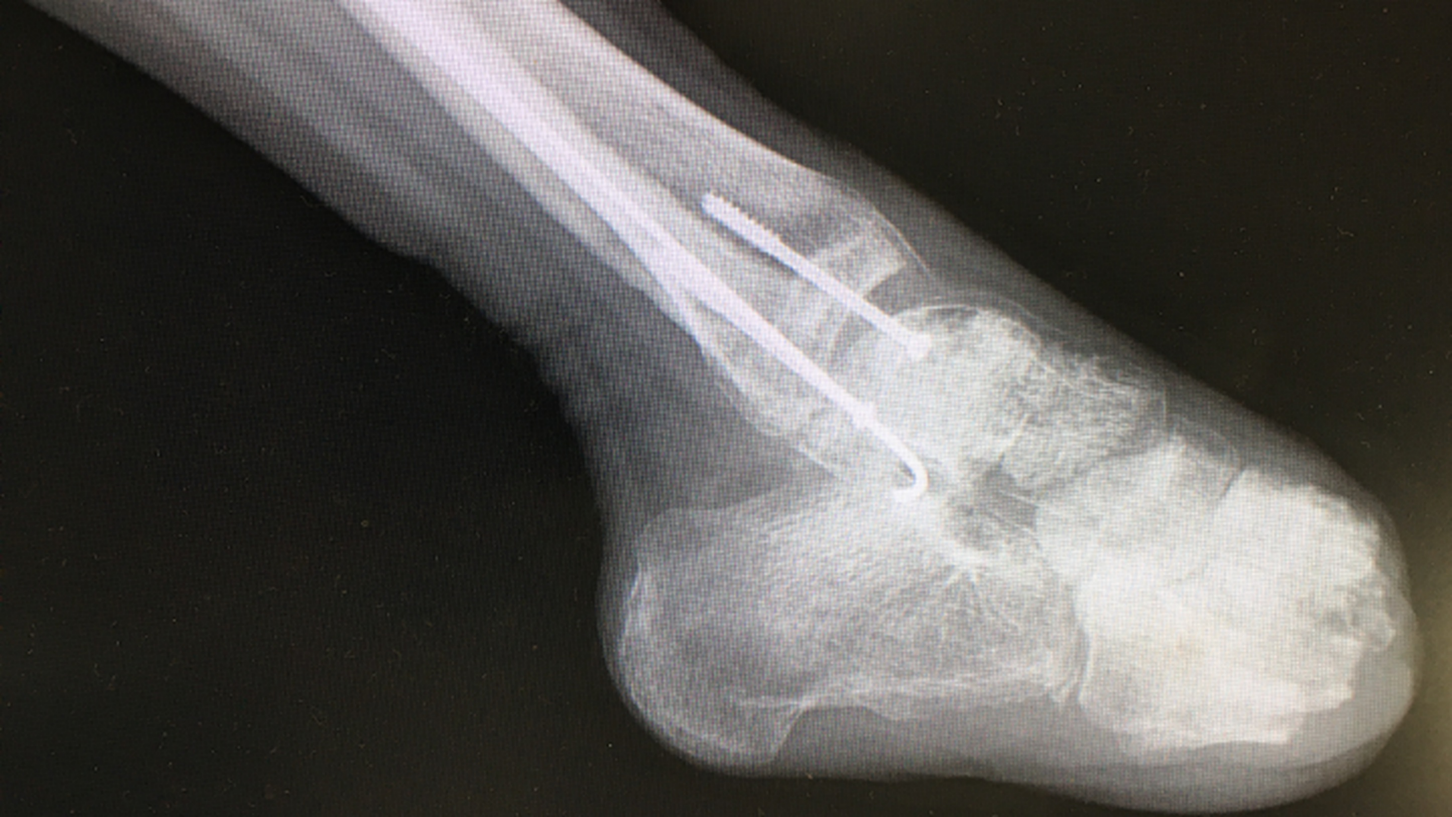
Plain radiograph of the foot at initial presentation Radiograph reveals significant osteopenia and angular deformity of the ankle joint, indicating advanced structural compromise.

He was referred to our hospital six months after the initial injury. At that time, the left foot had been amputated at the metatarsal level, with persistent drainage from a sinus tract (Figure 1). The ankle was fixed in approximately −45° dorsiflexion and 55° plantarflexion, and radiographs demonstrated marked osteopenia (Figure 2). Following multidisciplinary counseling, the patient elected to undergo transtibial amputation, which was performed 6.5 months after the initial injury.

At 7.5 months after the initial injury, the patient was admitted for prosthetic rehabilitation. The residual limb measured 18 cm in length (approximately 50% of the contralateral side). Manual muscle testing demonstrated normal muscle strength around the hip and knee joints on the amputated side, with no evidence of contracture. At this stage, mobility was independent with the use of a wheelchair or crutches.

Prosthetic prescription included a silicone liner with pin suspension, a total surface bearing (TSB) socket, and a Proflex-XC foot (Össur, Reykjavik, Iceland). Initial prosthetic rehabilitation was conducted according to the standard program routinely implemented at our institution [16]. The rehabilitation program consisted of 40 minutes per day of supervised physical therapy in combination with structured self-directed training during non-therapy hours, averaging 3-4 hours per day. Within eight weeks, the patient achieved independent level walking, stair climbing, and outdoor ambulation (Video 1).

**Video 1:** Level-ground ambulation with a prosthesis.

Following the achievement of independent basic ambulation, advanced high-impact training was introduced in accordance with the patient’s goal of returning to physically demanding work and sports. The program included squats, rope skipping, and unilateral hopping on the prosthetic side. These exercises were initially performed under a physiotherapist's supervision to ensure safety and appropriate technique, after which the patient continued them as self-directed training (Video 2).

**Video 2:** Unilateral hopping exercise performed on the prosthetic side.

The patient was discharged home after four months of inpatient rehabilitation and continued self-directed training at least twice weekly. Six months after discharge, he successfully returned to his pre-injury occupation, and at one year post-discharge, he commenced participation in competitive para-snowboarding (Video 3).

**Video 3:** Snowboarding on the prosthetic limb using the same device as for daily ambulation.

**Figure 3:**
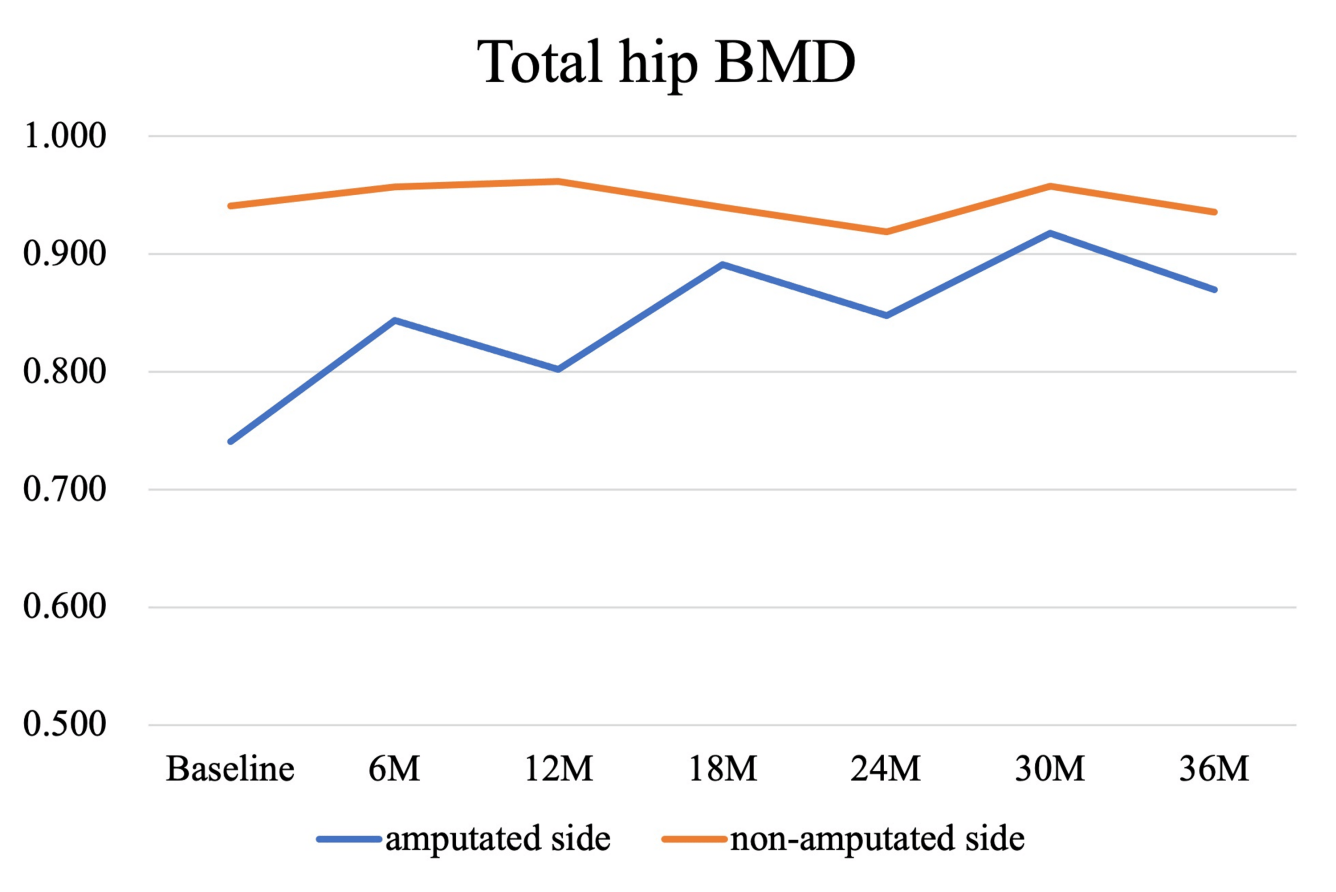
Longitudinal changes in BMD at the total hip over 36 months The vertical axis represents bone mineral density (BMD) values in grams per square centimeter (g/cm²). At baseline, the BMD on the amputated side was 78.7% of that on the non-amputated side. However, a progressive improvement was observed over time, with a 17.4% increase from baseline at 36 months. At that time point, the BMD of the amputated side reached 92.9% of the value on the non-amputated side.

**Figure 4:**
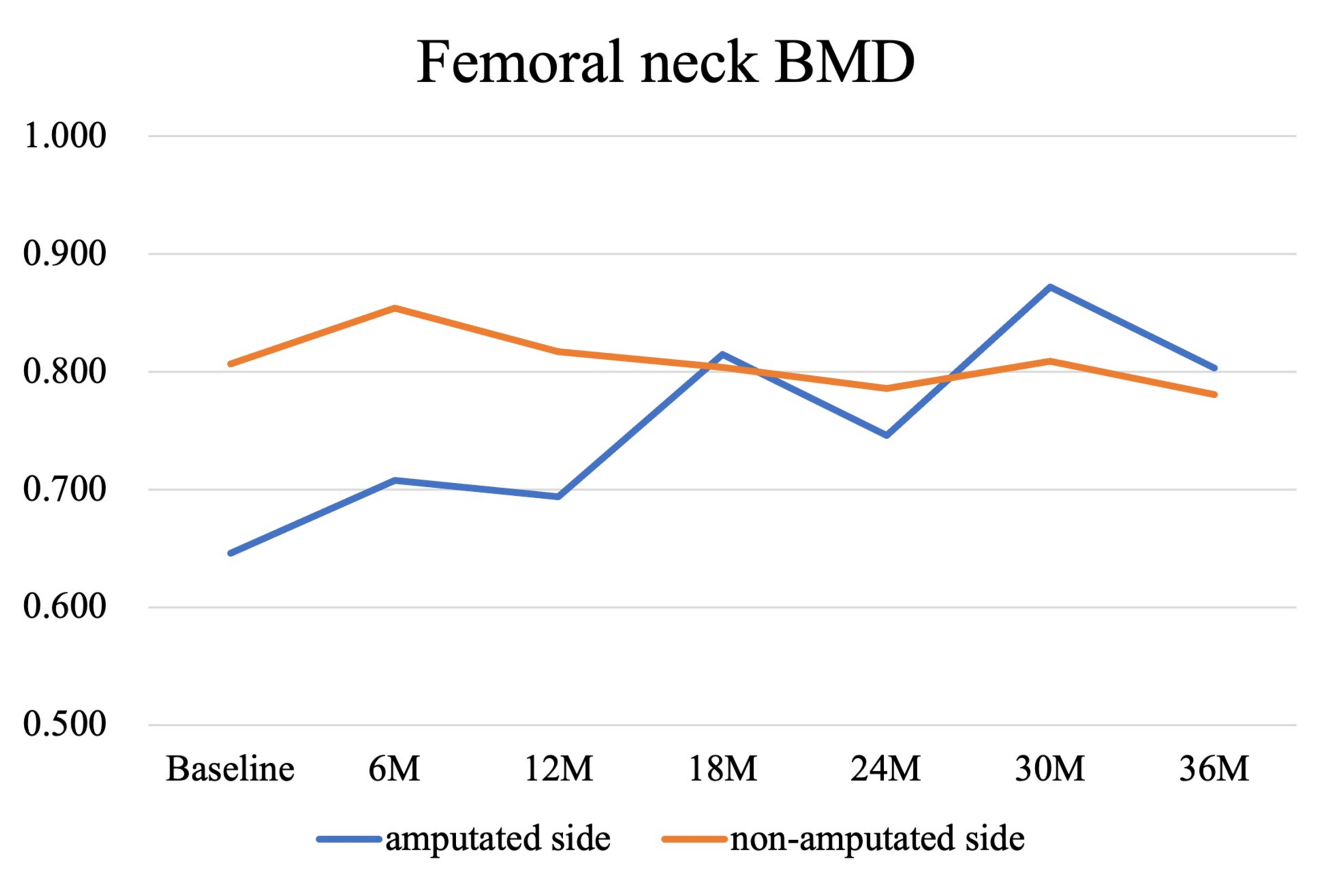
Longitudinal changes in BMD at the femoral neck over 36 months At baseline, the bone mineral density (BMD) on the amputated side was 80.0% of that on the non-amputated side. Over time, BMD increased progressively, reaching a 24.3% improvement from baseline at 36 months. At that time point, the BMD of the amputated side exceeded that of the non-amputated side, reaching 102.8%.

**Figure 5:**
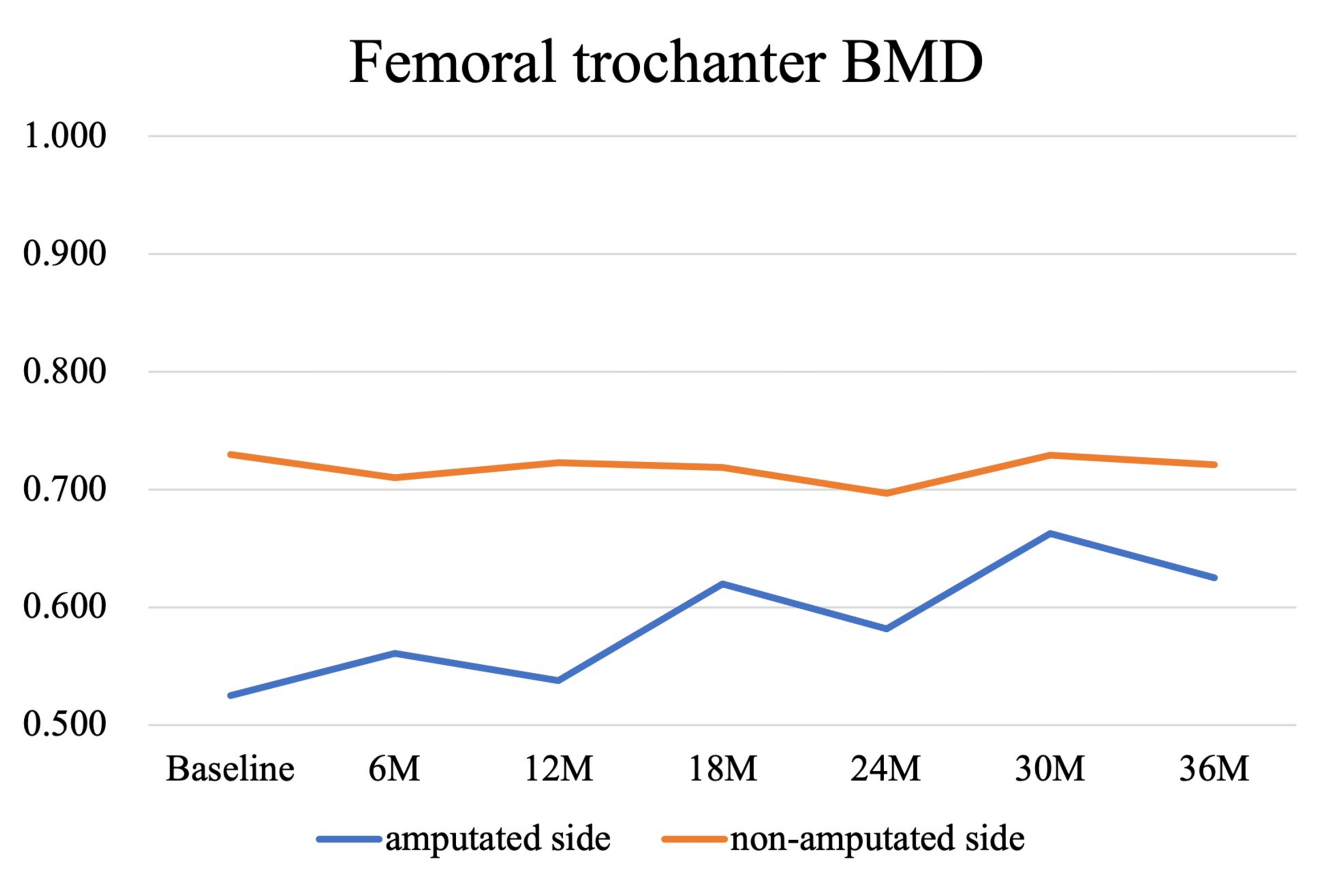
Longitudinal changes in BMD at the femoral trochanter over 36 months The bone mineral density (BMD) on the amputated side was initially 71.9% of that on the non-amputated side. However, a gradual improvement was observed over time, with a 19.0% increase from baseline at 36 months. At that time point, the BMD of the amputated side reached 86.7% of the value on the non-amputated side.

**Figure 6:**
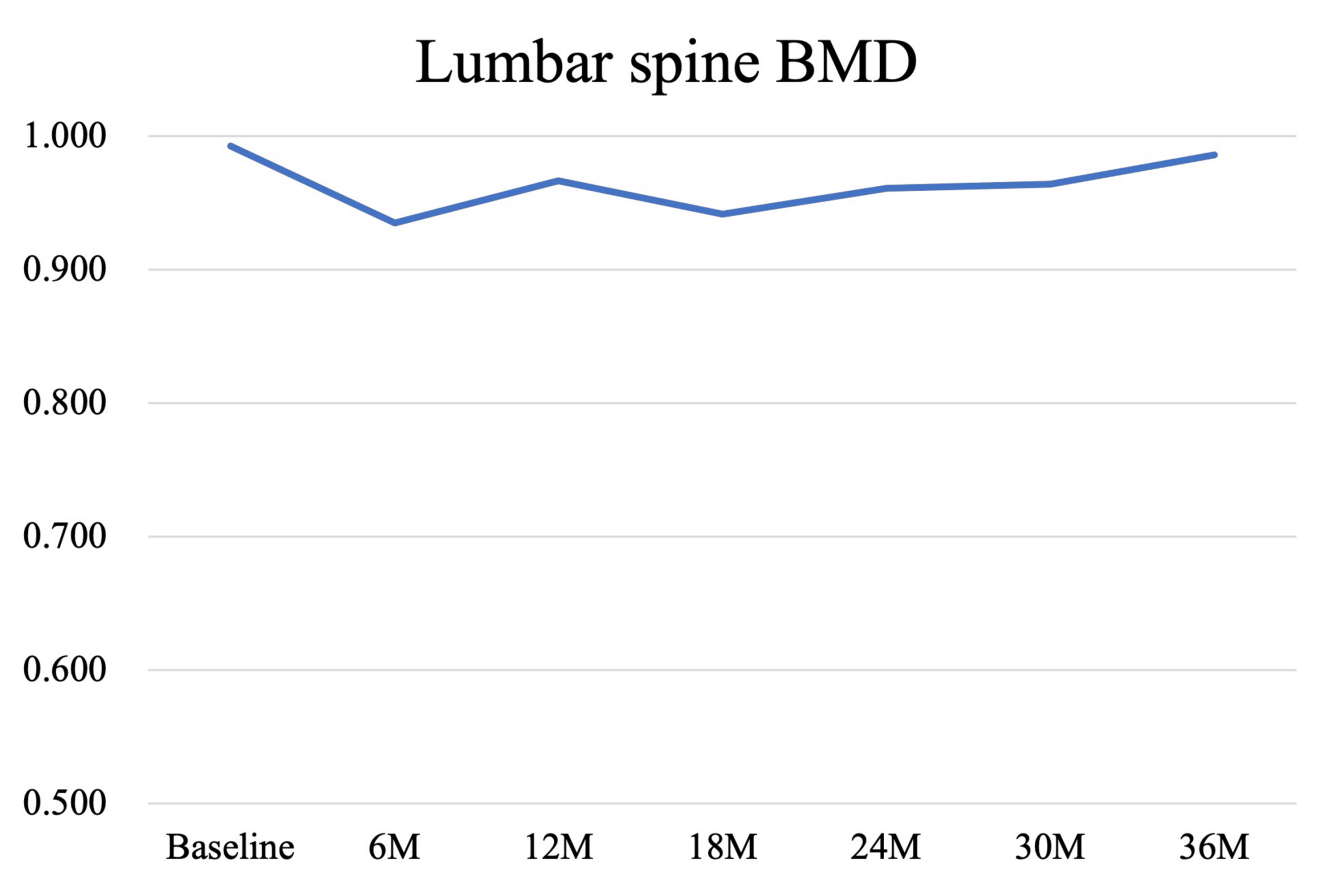
Longitudinal changes in BMD at the lumbar spine over 36 months Although an initial decline in bone mineral density (BMD) was observed, values gradually recovered and approached baseline levels by the 36-month follow-up. At that time point, lumbar spine BMD remained 0.7% below the baseline value.

**Figure 7:**
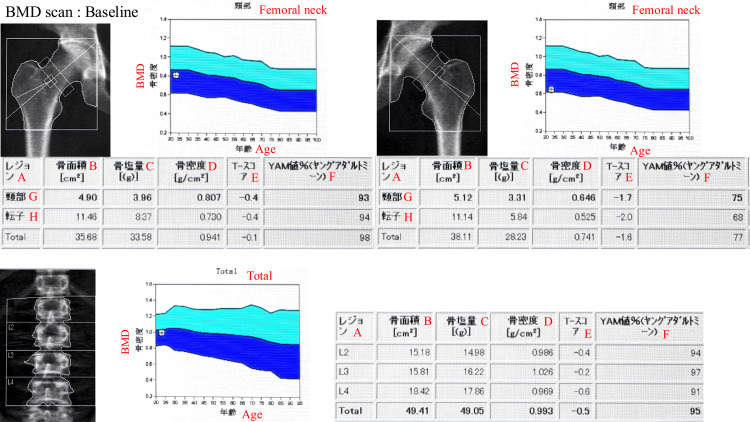
DXA scan data at baseline The upper left image represents the right hip (non-amputated side), the upper right image represents the left hip (amputated side), and the lower image shows the lumbar spine. The red annotations were added to indicate the graph legend. In each graph, the light blue area corresponds to the Z-score range between 0 and +2.0 standard deviations (SD), and the blue area corresponds to the range between 0 and −2.0 SD. The cross symbols (+) plotted within the graph indicate the patient’s measured BMD values. The accompanying table (originally in Japanese) includes the following parameters: A, measurement region; B, bone area (cm²); C, bone mineral content (BMC, g); D, bone mineral density (BMD, g/cm²); E, T-score; F, %YAM (Young Adult Mean percentage); G, femoral neck region; H, femoral trochanter region. All personally identifiable information has been removed from the scan data to ensure full anonymity and compliance with research ethics.

**Figure 8:**
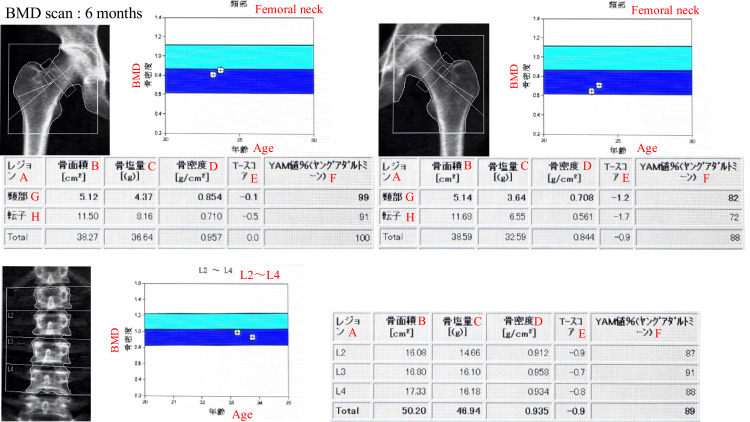
DXA scan data at 6 months The upper left image represents the right hip (non-amputated side), the upper right image represents the left hip (amputated side), and the lower image shows the lumbar spine. The red annotations were added to indicate the graph legend. In each graph, the light blue area corresponds to the Z-score range between 0 and +2.0 SD, and the blue area corresponds to the range between 0 and −2.0 SD. The cross symbols (+) plotted within the graphs indicate the longitudinal changes in BMD at each measurement site. The accompanying table (originally in Japanese) includes the following parameters: A, measurement region; B, bone area (cm²); C, bone mineral content (BMC, g); D, bone mineral density (BMD, g/cm²); E, T-score; F, %YAM (Young Adult Mean percentage); G, femoral neck region; H, femoral trochanter region. All personally identifiable information has been removed from the scan data to ensure full anonymity and compliance with research ethics.

**Figure 9:**
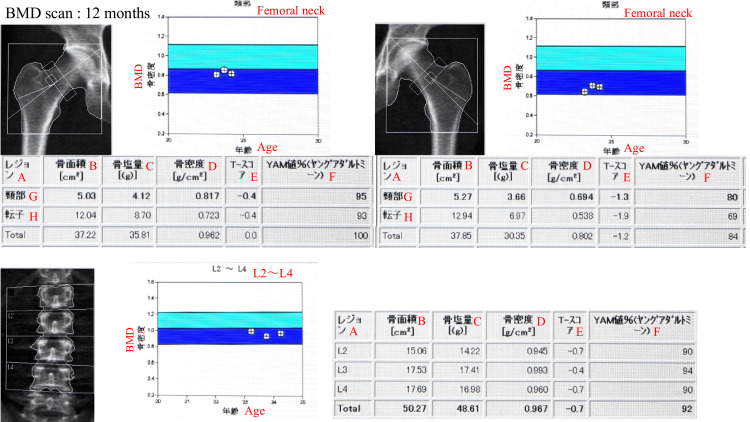
DXA scan data at 12 months The upper left image represents the right hip (non-amputated side), the upper right image represents the left hip (amputated side), and the lower image shows the lumbar spine. The red annotations were added to indicate the graph legend. In each graph, the light blue area corresponds to the Z-score range between 0 and +2.0 SD, and the blue area corresponds to the range between 0 and −2.0 SD. The cross symbols (+) plotted within the graphs indicate the longitudinal changes in BMD at each measurement site. The accompanying table (originally in Japanese) includes the following parameters: A, measurement region; B, bone area (cm²); C, bone mineral content (BMC, g); D, bone mineral density (BMD, g/cm²); E, T-score; F, %YAM (Young Adult Mean percentage); G, femoral neck region; H, femoral trochanter region. All personally identifiable information has been removed from the scan data to ensure full anonymity and compliance with research ethics.

**Figure 10:**
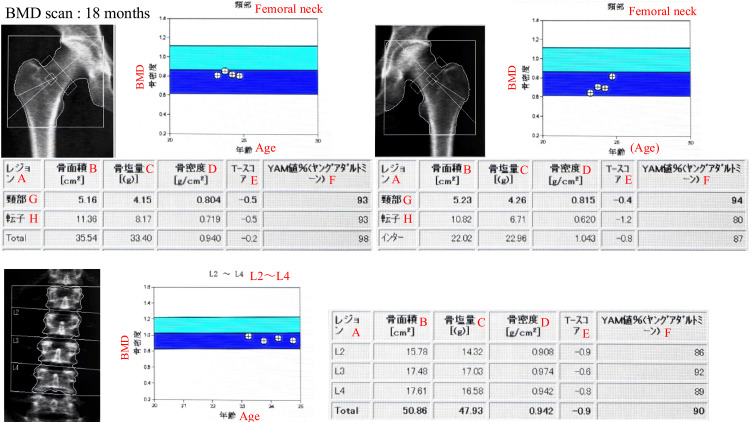
DXA scan data at 18 months The upper left image represents the right hip (non-amputated side), the upper right image represents the left hip (amputated side), and the lower image shows the lumbar spine. The red annotations were added to indicate the graph legend. In each graph, the light blue area corresponds to the Z-score range between 0 and +2.0 SD, and the blue area corresponds to the range between 0 and −2.0 SD. The cross symbols (+) plotted within the graphs indicate the longitudinal changes in BMD at each measurement site. The accompanying table (originally in Japanese) includes the following parameters: A, measurement region; B, bone area (cm²); C, bone mineral content (BMC, g); D, bone mineral density (BMD, g/cm²); E, T-score; F, %YAM (Young Adult Mean percentage); G, femoral neck region; H, femoral trochanter region. All personally identifiable information has been removed from the scan data to ensure full anonymity and compliance with research ethics.

**Figure 11:**
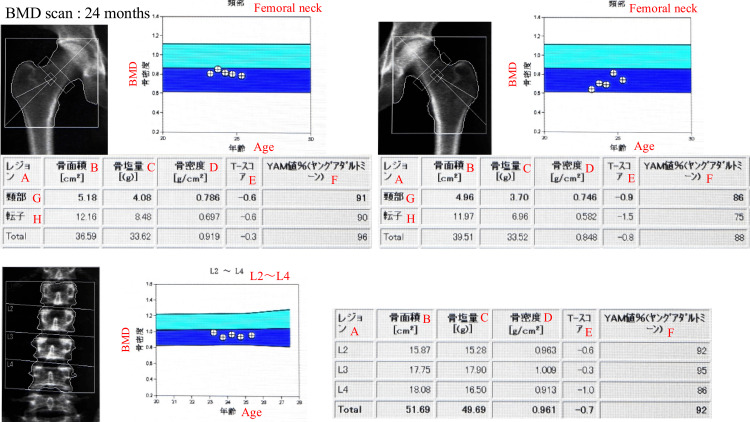
DXA scan data at 24 months The upper left image represents the right hip (non-amputated side), the upper right image represents the left hip (amputated side), and the lower image shows the lumbar spine. The red annotations were added to indicate the graph legend. In each graph, the light blue area corresponds to the Z-score range between 0 and +2.0 SD, and the blue area corresponds to the range between 0 and −2.0 SD. The cross symbols (+) plotted within the graphs indicate the longitudinal changes in BMD at each measurement site. The accompanying table (originally in Japanese) includes the following parameters: A, measurement region; B, bone area (cm²); C, bone mineral content (BMC, g); D, bone mineral density (BMD, g/cm²); E, T-score; F, %YAM (Young Adult Mean percentage); G, femoral neck region; H, femoral trochanter region. All personally identifiable information has been removed from the scan data to ensure full anonymity and compliance with research ethics.

**Figure 12:**
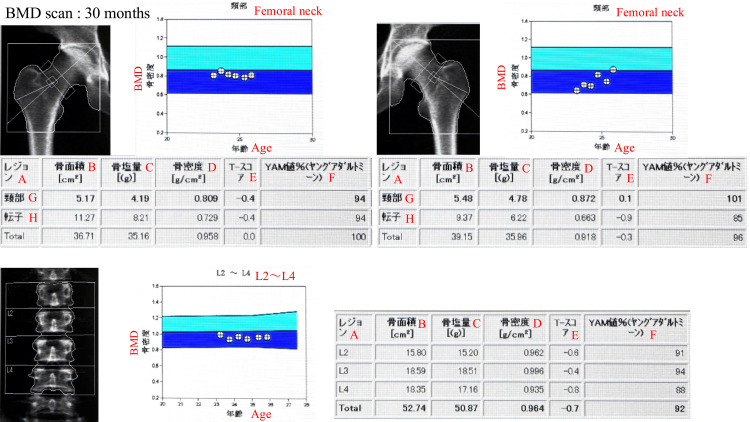
DXA scan data at 30 months The upper left image represents the right hip (non-amputated side), the upper right image represents the left hip (amputated side), and the lower image shows the lumbar spine. The red annotations were added to indicate the graph legend. In each graph, the light blue area corresponds to the Z-score range between 0 and +2.0 SD, and the blue area corresponds to the range between 0 and −2.0 SD. The cross symbols (+) plotted within the graphs indicate the longitudinal changes in BMD at each measurement site. The accompanying table (originally in Japanese) includes the following parameters: A, measurement region; B, bone area (cm²); C, bone mineral content (BMC, g); D, bone mineral density (BMD, g/cm²); E, T-score; F, %YAM (Young Adult Mean percentage); G, femoral neck region; H, femoral trochanter region. All personally identifiable information has been removed from the scan data to ensure full anonymity and compliance with research ethics.

**Figure 13:**
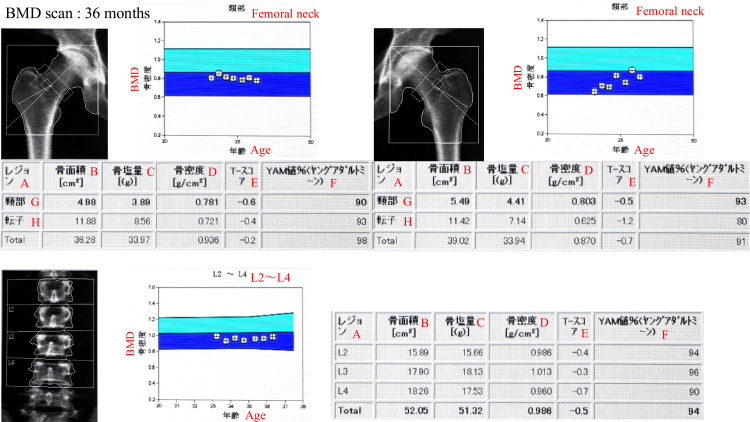
DXA scan data at 36 months The upper left image represents the right hip (non-amputated side), the upper right image represents the left hip (amputated side), and the lower image shows the lumbar spine. The red annotations were added to indicate the graph legend. In each graph, the light blue area corresponds to the Z-score range between 0 and +2.0 SD, and the blue area corresponds to the range between 0 and −2.0 SD. The cross symbols (+) plotted within the graphs indicate the longitudinal changes in BMD at each measurement site. The accompanying table (originally in Japanese) includes the following parameters: A, measurement region; B, bone area (cm²); C, bone mineral content (BMC, g); D, bone mineral density (BMD, g/cm²); E, T-score; F, %YAM (Young Adult Mean percentage); G, femoral neck region; H, femoral trochanter region. All personally identifiable information has been removed from the scan data to ensure full anonymity and compliance with research ethics.

Bone mineral density (BMD) measurements were performed in parallel with the rehabilitation program and post-discharge follow-up. Dual-energy X-ray absorptiometry (DXA) scans (Discovery, Hologic, MA, USA) were conducted at baseline, corresponding to the time of admission to our hospital (1 month post-amputation), and subsequently at 6, 12, 18, 24, 30, and 36 months, targeting the lumbar spine, total hip, femoral neck, and trochanter bilaterally. At baseline, BMD values of the amputated-side total hip, femoral neck, and trochanter were 78.7%, 80.0%, and 71.9% of the intact side, respectively. By the final assessment at 36 months, these values had increased by 17.4%, 24.3%, and 19.0%, reaching 92.9%, 102.8%, and 90.9% of the intact side. Lumbar spine BMD showed a 5% decline at 6 months but recovered to the baseline level by 36 months. These longitudinal changes are illustrated in Figures 3-6. The DXA scan data at each time point are provided in the Appendices as Figures 7-13.

## Discussion

This case demonstrates not only successful functional recovery following transtibial amputation and prosthetic rehabilitation but also progressive improvement in bone mineral density (BMD) over a three-year follow-up. Prior investigations have reported persistent deficits or, at best, maintenance of BMD, but not recovery in the residual limb. Indeed, longitudinal data remain scarce; Finco et al. described recovery of lumbar spine BMD to baseline levels within 12 months, while Hoellwarth et al. reported long-term reductions in hip BMD more than five years after amputation [17, 18]. In contrast, our case provides the first evidence of sustained improvement in amputated-side hip BMD over three years.

One possible explanation for this favorable outcome is the nature of the rehabilitation program. Evidence from non-amputee populations indicates that high-impact exercise plays a pivotal role in augmenting hip BMD, as demonstrated in postmenopausal women, elderly men, individuals with anorexia nervosa, and even astronauts under microgravity exposure [10-14]. These findings suggest that the favorable outcome in our patient may likewise be attributable to the osteogenic stimulus provided by intensive, impact-loading rehabilitation. Although implemented prior to the recent recommendations of Behan et al. [15], the patient engaged in a demanding training program, including squats, rope skipping, and unilateral hopping, that closely mirrors the progressive loading strategies now advocated to promote musculoskeletal adaptation. Taken together, this case highlights that structured, advanced prosthetic training may not only facilitate high-level functional recovery but also confer skeletal benefits to the amputated limb.

While the present case provides rare longitudinal evidence of hip BMD improvement following transtibial amputation, its interpretation should be contextualized within the broader literature. In non-amputee populations, controlled trials have consistently demonstrated that bone adaptation depends not merely on the presence of exercise, but on meeting specific thresholds of intensity, frequency, and duration [10-14]. In contrast, evidence in amputee cohorts remains largely descriptive, focusing on cross-sectional deficits in BMD rather than interventional outcomes [1-5]. Thus, our observation extends prior knowledge by suggesting that appropriately designed high-impact rehabilitation may counteract skeletal decline even under the altered loading conditions imposed by prosthesis use, yet the precise loading requirements remain to be elucidated.

A major limitation of the present case report is the inability to quantitatively assess the patient’s exercise load or training volume. Although functional recovery and improvements in bone mineral density (BMD) were observed, the precise relationship between the magnitude of mechanical loading and subsequent skeletal adaptation remains uncertain. Evidence from a randomized controlled trial in non-amputee populations has demonstrated that specific thresholds of exercise are necessary to elicit osteogenic responses, such as sufficiently high-intensity resistance training or structured impact-loading protocols [19]. However, whether such thresholds apply to individuals with lower limb amputation remains unclear, given the altered loading environment and the biomechanical consequences of prosthesis use. Future research should therefore aim to establish dose-response relationships between exercise parameters and BMD in amputees, with particular emphasis on the hip and spine, which are highly vulnerable to osteoporosis and fracture.

## Conclusions

This case demonstrates that structured prosthetic rehabilitation incorporating high-impact exercise can achieve not only advanced functional recovery but also sustained improvement in hip BMD following transtibial amputation. These findings provide preliminary clinical evidence that targeted, progressive loading strategies may mitigate skeletal decline in amputees, a population traditionally considered at high risk for osteoporosis and fracture. Although further research is required to define the optimal exercise parameters and confirm generalizability, this report highlights the potential of high-impact rehabilitation as an adjunct to preserve musculoskeletal health after limb loss.
